# Psychometric Properties of the Arabic Dissociative Symptoms Scale—Brief Across Five Arab Countries

**DOI:** 10.1002/puh2.70115

**Published:** 2025-09-18

**Authors:** Anthony Rizk, Adella Ibrahim, Diana Malaeb, Amira M. Ali, Mirna Fawaz, Nouran Omar El Said, Nisma Merdad, Rizwana Amin, Wizra Saeed, Muna Barakat, Rami Mosleh, Feten Fekih‐Romdhane, Souheil Hallit, Sahar Obeid

**Affiliations:** ^1^ School of Arts and Sciences Holy Spirit University of Kaslik Jounieh Lebanon; ^2^ College of Pharmacy Gulf Medical University Ajman UAE; ^3^ Department of Psychiatric Nursing and Mental Health, Faculty of Nursing Alexandria University Alexandria Egypt; ^4^ Faculty of Health Sciences, Nursing Department Beirut Arab University Beirut Lebanon; ^5^ Department of Pharmacy Practice and Clinical Pharmacy, Faculty of Pharmacy Future University in Egypt Cairo Egypt; ^6^ Psychology Department, College of Humanities Effat University Jeddah Saudi Arabia; ^7^ Department of Clinical Pharmacy and Therapeutics, Faculty of Pharmacy Applied Science Private University Amman Jordan; ^8^ Department of Clinical and Community Pharmacy, Faculty of Pharmacy An‐Najah National University Nablus Palestine; ^9^ The Tunisian Center of Early Intervention in Psychosis, Department of Psychiatry “Ibn Omrane” Razi Hospital Manouba Tunisia; ^10^ Faculty of Medicine of Tunis Tunis El Manar University Tunis Tunisia; ^11^ School of Medicine and Medical Sciences Holy Spirit University of Kaslik Jounieh Lebanon; ^12^ Department of Psychology and Education, School of Arts and Sciences Lebanese American University Jbeil Lebanon

**Keywords:** Arabic population, Dissociative Symptoms Scale—Brief, psychometric properties

## Abstract

**Background:**

Dissociation, involving disruptions in cognition, perception, and identity, is closely linked to trauma and various psychiatric disorders but remains underrecognized, especially in non‐Western contexts. Although tools like the Dissociative Symptoms Scale—Brief (DSS—B) have improved assessment, validated Arabic‐language versions are lacking. Given rising mental health concerns and limited resources in the Arab world, this study aims to evaluate the psychometric properties of the Arabic‐translated DSS—B to support culturally appropriate diagnosis and research on dissociation.

**Methods:**

In this cross‐sectional study, participants from KSA, Egypt, Lebanon, Palestine, and Jordan were recruited via snowball sampling and completed an online survey. The DSS—B was translated into Arabic using a forward‐backward method and reviewed by experts for cultural and semantic accuracy. Participants also completed validated Arabic versions of the Jong‐Gierveld Loneliness Scale, Patient Health Questionnaire‐4, and the Brief Irritability Test.

**Results:**

Among 1494 participants (mean age = 24.97; 74.5% female), Palestinians showed the highest dissociative symptoms. Confirmatory factor analysis confirmed good model fit, excellent reliability (*ω* = 0.93; *α* = 0.92), and strong convergent validity average variance extracted (AVE = 0.70). Measurement invariance across genders and countries was supported, with no significant gender differences in scores. Dissociation was positively correlated with depression‐anxiety (*r* = 0.57), irritability (*r* = 0.51), and loneliness (*r* = 0.45), confirming concurrent validity, while discriminant validity was also established.

**Conclusion:**

This study validates the Arabic DSS—B as a reliable, valid, and culturally adaptable tool for assessing dissociation in Arab populations, reinforcing its clinical and research utility. Future research should explore its generalizability in underrepresented groups, use longitudinal and clinician‐based assessments, and investigate neurobiological underpinnings to deepen understanding and application of dissociation measurement globally.

## Graphical abstract

This study validated the Arabic Dissociative Symptoms Scale—Brief (DSS—B) across five Arab countries, demonstrating excellent reliability and cultural relevance. The tool enables accurate assessment of dissociation in Arabic‐speaking populations for both clinical practice and research.

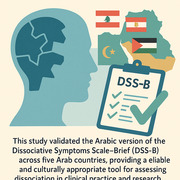

## 1 Introduction

Dissociation is a central concept in modern psychopathology, encompassing a disruption or disconnection in the normal integration of cognitive and psychological functions such as consciousness, memory, identity, emotion, perception, body representation, motor control, and behavior [1]. These disruptions exist on a continuum, ranging from mild, everyday experiences—such as becoming fully engrossed in a book or movie—to more severe and distressing states, including depersonalization, where individuals feel detached from their own body or thoughts, and derealization, where the external world appears unreal or distorted [2]. The Diagnostic and Statistical Manual of Mental Disorders‐Fifth Version (DSM‐5) formally recognizes several dissociative disorders, including dissociative amnesia, depersonalization/derealization disorder, and dissociative identity disorder, each of which manifests through distinct yet interrelated symptoms [1, 3].

A strong association has been established between dissociation and trauma, particularly early‐life or cumulative traumatic experiences. Dissociative responses during trauma—collectively referred to as *peritraumatic dissociation*—such as tunnel vision, trance‐like states, confusion, temporal disorientation, and amnesia, have been strongly linked to the subsequent development of posttraumatic stress disorder (PTSD) [4]. Dissociative symptoms are strongly connected with trauma exposure, particularly in cases of early childhood adversity and disruptions in attachment and caregiving. Thus, research highlights dissociation as a significant yet often overlooked factor linking trauma to PTSD and various health and social challenges [5].

The DSM‐5 acknowledges this connection by introducing a dissociative subtype of PTSD (PTSD‐DS), characterized by depersonalization and derealization symptoms triggered by reminders of past trauma [1]. Nevertheless, research overwhelmingly refutes this notion, demonstrating that dissociative disorders are both prevalent and clinically significant, affecting individuals across diverse populations [4].

Dissociative symptoms extend beyond trauma‐related disorders and have been implicated in a wide range of psychiatric conditions, including borderline personality disorder, schizophrenia, eating disorders, panic disorder, affective disorders, and obsessive‐compulsive disorder. In these conditions, dissociation is associated with increased symptom severity, functional impairment, and difficulties in emotion regulation [2, 6, 7]. Moreover, it has been identified as a predictor of poor treatment response, particularly in psychotherapeutic interventions for PTSD, OCD, and panic disorder, where individuals exhibiting dissociative symptoms often show reduced improvement compared to those without such symptoms [2, 8, 9].

Furthermore, dissociation is closely linked to various mental health conditions, including anxiety and depression. Dissociative symptoms and disorders have been strongly and directly associated with anxiety and depression [10], highlighting their significant connection to these psychological conditions [11]. Furthermore, research has identified both direct and indirect correlations between dissociation and other emotional states, such as loneliness and irritability [12], suggesting that dissociative experiences may contribute to or be influenced by a broader range of psychological distress [13]. Additionally, it may contribute to paranoia, grandiosity, and cognitive disorganization [10]. Conceptually, dissociation can be understood as a paradoxical mechanism—whereas it serves as a coping strategy to distance individuals from distressing memories, it simultaneously contributes to long‐term psychological maladjustment and emotional dysregulation [14].

Dissociation and dissociative disorders have regained attention, especially in North America, prompting new research approaches [15]. Techniques like network analysis gained popularity in 2019 [16, 17], and a 2020 meta‐analysis linked dissociation to psychotic symptoms [18]. Despite progress, more research is needed to refine diagnostics and interventions [10, 15]. Reliable assessment tools like the Dissociative Symptoms Scale—Brief (DSS—B) support this effort [19, 20].

Dissociative disorders remain a frequently overlooked area in clinical practice, despite their profound impact on daily functioning, mental health, and overall well‐being [4]. These disorders are closely linked to chronic suicidality and self‐destructive behaviors, emphasizing the urgent need for a multidimensional and integrative approach in psychiatric and psychological care [5]. Furthermore, dissociation is not merely a pathological construct but a complex and adaptive phenomenon that interacts with the broader field of psycho‐traumatology. The evolving nature of dissociation research, particularly in emerging areas such as cyber dissociation, highlights the need for continuous exploration and refinement of our understanding of dissociative processes [5]. However, despite these advancements, dissociation remains underrepresented in mental health training programs, leaving many clinicians and researchers with misconceptions shaped more by media portrayals than by scientific literature [4, 5]. To mitigate the impact of dissociation and its associated social costs, it is essential to identify effective interventions for individuals experiencing dissociative symptoms [21]. Despite theories suggesting that dissociation may be present across various psychiatric disorders, affecting both their symptoms and treatment outcomes, it remains widely under‐recognized. This challenge is further exacerbated by the lack of clear understanding of dissociation's role in mental health [10].

### 1.1 Dissociative Symptoms Scale

Dissociative symptoms represent a fundamental aspect of dissociative disorders and other psychiatric conditions, making diagnosis particularly challenging [22]. Consequently, the availability of valid, reliable, and user‐friendly tools for assessing dissociation is crucial for advancing knowledge and improving clinical practice. Various instruments have been created to measure dissociation, regardless of its specific definition [23]. A systematic review identified 44 different tools used across 170 studies, including the Dissociative Ability Scale (DAS), the Depersonalization Severity Scale, the Child/Adolescent Dissociation Checklist (CADC), the Clinician‐Administered Dissociative States Scale (CADSS), the Detachment and Compartmentalization Inventory (DCI), and the Depersonalization‐Derealization Inventory (DDI) [20].

One such tool, the Dissociative Symptoms Scale (DSS), plays a vital role in improving the identification and management of dissociative conditions. Developed in the United States, the DSS is a 20‐item self‐report instrument designed to assess moderate‐to‐severe levels of depersonalization, derealization, memory gaps, and dissociative re‐experiencing across various clinical populations. Its use contributes significantly to enhancing awareness, detection, and treatment of dissociative disorders [5, 24]. The DSS has also been translated into Italian and validated in a mixed clinical and nonclinical sample, demonstrating strong psychometric properties [25]. Findings supported its reliability and validity among Italian adult outpatients and community members, with a Cronbach's *α* of 0.89, aligning with Carlson's research [25].

Among the many dissociation measures, the Dissociative Experiences Scale (DES) stands out as the most widely utilized and extensively evaluated tool, receiving strong ratings for internal consistency and reliability [20, 26]. Initially a 28‐item self‐report questionnaire, the DES is comprised of three subscales: amnestic identity fragmentation, absorption and imaginative involvement, and depersonalization/derealization [27]. It was later refined into the DES‐II [28].

Despite its widespread application, the DES has notable limitations, such as item misfit, variability in mean scores, and a tendency for respondents to select lower response categories [29, 30, 31]. Additionally, its Arabic translation yielded a reliability score below the internationally preferred threshold (Cronbach's *α* = 0.67; [32]). In response to these challenges, a concise, practical, and time‐efficient eight‐item measure, the DSS—B, was developed and validated. It has been translated by the same author and same article into English and Spanish, with psychometric properties assessed in both languages [19]. Reliability was strong, with categorical omega estimates of 0.886 for the validation sample. Temporal stability was moderate, with Spearman correlations of 0.664 and 0.574 in two samples. Validity testing showed significantly higher scores in clinical and PTSD samples. Measurement invariance was supported across ethnoracial groups, with partial invariance between White/European American and Asian American groups and full invariance between Black/African American and Hispanic/Latin American groups. Confirmatory factor analysis confirmed the factor structure in both language versions [19].

### 1.2 The Present Study

Mental health issues have been increasing in the Arab world at rates higher than the rest of the world [33]. In response, the amount of research on mental health in Arab countries has grown substantially over the last 10 years, increasing by about 160% in 22 Arab countries compared to 57% globally [34, 35]. Nevertheless, even with this progress, the Arab world still accounts for only 2% of worldwide mental health research, despite compromising 6% of the world's population. Common conditions, such as depression, schizophrenia, anxiety, PTSD, bipolar disorder, ADHD, and autism, have been the main focus of research in the region. Many psychiatric disorders, such as PTSD, anxiety, depression, and psychotic symptoms, are closely linked to dissociation [1, 10, 15]. However, because its symptoms overlap with those of other mental health problems, it is still a challenging illness to identify [22, 32]. It is crucial to create and validate psychometrically sound tools in order to improve our comprehension and detection of dissociation. Although interest in dissociation research has grown, standardized Arabic‐language measures remain scarce. Such validated instruments could facilitate research on dissociative behaviors and their subcategories, improving understanding within a local context. Given the increasing global focus on dissociation, promoting research in Arab populations has become imperative. Therefore, this study aims to evaluate the psychometric properties of the Arabic‐translated DSS—B among Arabic‐speaking adults.

## 2 Methods

### 2.1 Minimum Sample Size Calculation

We estimated a minimum sample of 160 participants based on the recommendation of 20 times per scale's variables [36].

### 2.2 Participants

Data were collected through Google Forms between December 2024 and January 2025. Arabic‐speaking participants from KSA, Egypt, Lebanon, Palestine, and Jordan were asked to fill the survey. We employed the snowball sampling technique, whereby participants are asked to spread the Google form link to people they know, and then these people spread it to their friends. After providing digital informed consent, participants were permitted to fill out the relevant scales in the Google Form. Participation in our study was completely anonymous, confidential, and voluntary. The data were securely stored in the principal's investigator's Google account, which is protected by authentication credentials, including a unique username and password, ensuring restricted access.

### 2.3 Translation Procedure

Before being used in this study, the DSS scale was translated and culturally adapted to fit the Arabic language and context. This process ensured semantic consistency between the original and Arabic versions, following international standards and guidelines [37]. The translation followed a forward and backward method: A Lebanese translator not involved in the study first translated the scale from English to Arabic, and then a Lebanese psychologist fluent in English retranslated it back into English. This method aimed to balance direct and contextual translations. A panel of experts, including two psychiatrists, one psychologist, the research team, and the translators, reviewed both the original and retranslated English versions to resolve any discrepancies and ensure accuracy [38]. The scale was also adapted to the study context to clarify ambiguities and facilitate interpretation, maintaining its conceptual integrity in both the original and Arabic contexts [39]. A pilot test confirmed the clarity of the questions, and no further modifications were required.

### 2.4 Questionnaire

Participants in our study provided their age, sex, country of birth, type of major they are studying, and the household crowding index (HCI), which is calculated by dividing the total count of people living in a household, excluding a newborn child, by the total number of rooms in that household, excluding the kitchen [40]. Additionally, participants were prompted answer the following scales.

#### 2.4.1 Jong‐Gierveld Loneliness Scale

The modified Jong‐Gierveld Loneliness Scale, which consists of five questions (e.g., “I experience a general sense of emptiness” and “I miss having people around”), was used to measure subjective loneliness. A “yes” response receives a score of 1, and a “no” response receives a score of 0. Scores for each item are added to calculate the instrument's final score. Higher scores suggest a greater sense of being lonely [41, 42]. This scale has been validated in Lebanon, and the validated Arabic version was used [43] (*ω* = 0.78/*α* = 0.78).

#### 2.4.2 Patient Health Questionnaire (PHQ‐4)

The PHQ‐4 is a concise four‐item questionnaire designed to assess anxiety and depressive symptoms experienced over the past 2 weeks [44]. It comprises two subscales: anxiety (e.g., “Feeling nervous, anxious or on edge”) and depression (e.g., “Little interest or pleasure in doing things”), each consisting of two items. Each item is rated on a 4‐point Likert scale ranging from 0 (not at all) to 3 (nearly every day). To calculate the total PHQ‐4 score, the scores from all four items are summed. The cutoff score for the PHQ‐4's subscales is greater than or equal to 3; a score of 0–2 indicates the absence of psychological distress, between 3 and 5 suggests mild psychological distress, between 6 and 8 suggests moderate psychological distress, and between 9 and 12 suggests severe psychological distress. This scale has been validated in Lebanon, and the validated Arabic version was used [45] (*ω* = 0.90/*α* = 0.90).

#### 2.4.3 The Brief Irritability Test (BITe)

The BITe is a five‐item self‐report tool that assesses irritability over the last 2 weeks [46]. Each item is rated on a 6‐point Likert scale ranging from 1 (never) to 6 (always). A total score is calculated by summing the score of each item, with higher scores reflecting more irritability, depicting the level of irritability of a subject. This scale has been validated in Lebanon, and the validated Arabic version was used [47] (*ω* = 0.93/*α* = 0.92).

### 2.5 Analytic Strategy

No missing data were found in the database because all questions were required in the Google Forms. We conducted a CFA via the SPSS AMOS v.30 software. The maximum likelihood method was used to obtain parameter estimates. We expected to replicate the four‐factor structure of the original scale. Multiple fit indices were calculated: root mean square error of approximation (RMSEA) (≤0.08), standardized root mean square residual (SRMR) (≤0.05), Tucker‐Lewis Index (TLI), and comparative fit index (CFI) (≥0.90 for both) [48]. Additionally, convergent validity was checked via the average variance extracted (AVE) ≥ 0.50 [49]. Multivariate normality was not verified at first (Bollen‐Stine bootstrap *p* = 0.002); therefore, we performed non‐parametric bootstrapping procedure.

A multi‐group CFA was conducted to examine measurement invariance of dissociative symptoms scores between genders [50]; we followed several steps to conclude to invariance: (a) configural invariance, (b) weak invariance (loadings), (c) strong invariance (loadings, intercepts), (d) strict invariance (loadings, intercepts, uniqueness), (e) invariance of the latent variance–covariance (loadings, intercepts, uniqueness, variance–covariance), and (f) latent means invariance (loadings, intercepts, uniqueness, variance–covariance, latent means) [51]. ΔCFI ≤ 0.010 and ΔRMSEA ≤ 0.015 or ΔSRMR ≤ 0.010 supported the evidence of invariance [52]. The comparison of dissociative symptoms scores between genders was done using the Mann–Whitney test and between countries using the Kruskal–Wallis test.

Internal reliability was assessed using McDonald's *ω* and Cronbach's *α*, with values greater than 0.70 reflecting adequate composite reliability [53]. The dissociative symptoms scores were not normally distributed as shown by skewness and kurtosis values outside the −1 and +1 interval [54]. The association between the HP scores and other scores was evaluated using the Spearman test.

## 3 Results

### 3.1 Sample's Characteristics

A total of 1494 participants filled the survey from five different countries, with a mean age of 24.97 years and 74.5% females. The description by country can be found in Table 1.

**TABLE 1:** Characteristics of the sample.

	Total (*n* = 1494)	Lebanon (*n* = 389)	Jordan (*n* = 308)	Egypt (*n* = 298)	Saudi Arabia (*n* = 282)	Palestine (*n* = 217)
Age (years)	24.97 ± 9.09	22.65 ± 7.26	26.71 ± 10.62	26.05 ± 8.55	27.63 ± 10.15	21.71 ± 6.76
Gender						
Male (%)	381 (25.5)	118 (30.3)	78 (25.3)	84 (28.2)	52 (18.4)	49 (22.6)
Female (%)	1113 (74.5)	271 (69.7)	230 (74.7)	214 (71.8)	230 (81.6)	168 (77.4)
HCI	1.16 ± 0.52	1.16 ± 0.51	1.10 ± 0.49	1.23 ± 0.51	0.96 ± 0.51	1.40 ± 0.52
Dissociation	4.53 ± 6.21	4.32 ± 6.10	3.72 ± 5.79	3.85 ± 6.02	4.59 ± 6.14	6.88 ± 6.81

Abbreviation: HCI, household crowding index.

### 3.2 Confirmatory Factor Analysis

**FIGURE 1:**
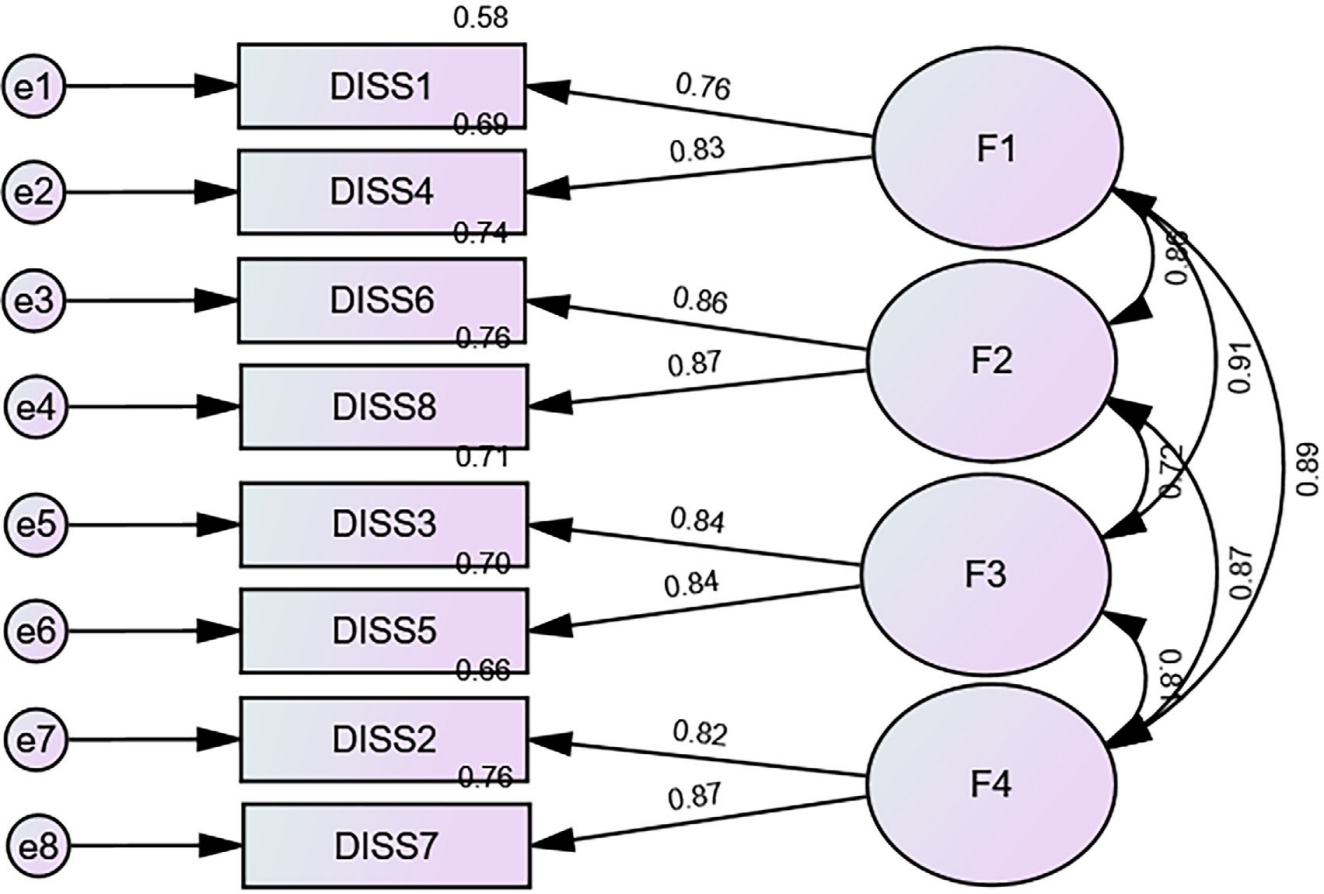
Standardized loading factors deriving from the confirmatory factor analysis of the four‐factor model of the dissociation scale—short form.

The fit indices of the one‐factor model were acceptable (*χ* ^2^/df = 64.71/14 = 4.62; RMSEA = 0.049 (90% CI 0.037, 0.062), SRMR = 0.014, CFI = 0.994, TLI = 0.987). The fit indices of the second‐order model were good as well: (*χ* ^2^/df = 153.74/16 = 9.61; RMSEA = 0.076 (90% CI 0.065, 0.087), SRMR = 0.024, CFI = 0.983, TLI = 0.970). The standardized estimates of factor loadings were all adequate and are shown in Figure 1. Internal reliability was excellent for the total score (*ω* = 0.93/*α* = 0.92). The AVE value was 0.70, supporting convergent validity.

### 3.3 Measurement Invariance Across Genders

The values of ΔCFI ≤ 0.010, ΔRMSEA ≤ 0.015, and ΔSRMR ≤ 0.010 supported the evidence of invariance of the dissociation scale between genders at all levels (Table 2).

**TABLE 2:** Measurement invariance of the dissociation scale—short form.

Model	CFI	RMSEA	SRMR	Model comparison	ΔCFI	ΔRMSEA	ΔSRMR
**Model 1: Invariance across sex**							
Configural	0.991	0.041	0.024				
Metric	0.991	0.038	0.024	Configural vs. metric	<0.001	0.003	<0.001
Scalar	0.991	0.036	0.024	Metric vs. scalar	<0.001	0.002	<0.001
Strict	0.989	0.036	0.028	Strict vs. scalar	0.002	<0.001	0.004
Latent variance–covariance	0.987	0.036	0.037	Latent variance–covariance vs. strict	0.002	<0.001	0.009
Latent means invariance	0.987	0.036	0.037	Latent means invariance vs. latent variance–covariance	<0.001	<0.001	<0.001
**Model 2: Invariance across countries**							
Configural	0.970	0.050	0.024				
Metric	0.968	0.046	0.035	Configural vs. metric	0.002	0.004	0.011
Scalar	0.967	0.043	0.034	Metric vs. scalar	0.001	0.003	0.001
Strict	0.915	0.060	0.036	Strict vs. scalar	0.052	0.017	0.002
Latent variance–covariance	0.883	0.062	0.050	Latent variance–covariance vs. strict	0.032	0.002	0.014
Latent means invariance	0.883	0.062	0.050	Latent means invariance vs. latent variance–covariance	<0.001	<0.001	<0.001

Abbreviations: CFI, comparative fit index; RMSEA, root mean square error of approximation; SRMR, standardized root mean square residual.

### 3.4 Group Comparisons of Means

Following the establishment of scalar invariance, latent mean comparisons were conducted. Results showed no significant difference was found between females and males in terms of dissociative symptoms (4.66 ± 6.29 vs. 4.48 ± 6.19; *t*(1492) = 0.49; *p* = 0.764, Cohen's *d* = 0.029).

Invariance was not fully supported at strict and latent variance–covariance levels across countries (Table 2). A higher mean dissociative symptoms score was found in Palestinians (6.88 ± 6.81), followed by Saudis (4.59 ± 6.14), Lebanese (4.32 ± 6.10), Egyptians (3.85 ± 6.02), and Jordanians (3.72 ± 5.79) (*F*(4, 1489) = 10.34; *p* < 0.001; Cohen's *d* = 0.027).

### 3.5 Concurrent and Discriminant Validity

Higher dissociative symptoms scores were significantly associated with higher depression‐anxiety (*r* = 0.57; *p* < 0.001), irritability (*r* = 0.51; *p* < 0.001), and loneliness (*r* = 0.45; *p* < 0.001) (Table 3).

**TABLE 3:** Pearson correlation matrix between scores.

	1	2	3
1. Dissociation	1		
2. Depression‐anxiety	0.57^***^	1	
3. Irritability	0.51^***^	0.65^***^	1
4. Loneliness	0.45^***^	0.45^***^	0.35^***^

*** *p* < 0.001.

The square root of the AVE value was 0.84, which was higher than the correlations between the factors, indicating discriminant validity.

## 4 Discussion

The objective of this study was to assess the psychometric properties of the Arabic adaptation of the DSS—B across five Arabic‐speaking countries: Lebanon, Jordan, Egypt, Saudi Arabia, and Palestine. The study aimed to evaluate the scale's reliability, validity, and measurement invariance to ensure its applicability across different populations. The results demonstrated strong factorial structure, with excellent model fit indices confirming its structural integrity. Internal reliability was high (*ω* = 0.93, *α* = 0.92), and the scale showed strong convergent and discriminant validity, effectively measuring dissociation as a distinct construct. Measurement invariance analyses confirmed that the DSS—B functions equivalently across genders and countries, allowing for meaningful comparisons. No significant gender differences were found, but dissociation levels varied across countries, with Palestinian participants reporting the highest scores. These findings support the DSS—B as a reliable and valid tool for assessing dissociation in Arabic‐speaking populations and highlight the influence of sociopolitical factors on dissociative symptoms.

The study's findings offer strong evidence of the Arabic DSS—B's validity and reliability in these five nations. The scale's structural integrity was confirmed by its excellent fit in both one‐factor and second‐order models. Similar to Macia et al.’s [19] findings on strong factor structure, this study reveals that the DSS—B underscores the scale's capacity to maintain its core measurement properties even when reduced in length. The scale's good internal reliability (*ω* = 0.93, *α* = 0.92) indicates that it captures dissociation symptoms consistently across various groups, which is fitting with Macia et al.’s [19] findings, particularly when categorical omega was used (*ωC* = 0.886, *α* = 0.829). This suggests that both studies recognize the DSS—B as a reliable instrument for measuring dissociation consistently across different populations.

The DSS—B performs similarly across genders and nations, according to measurement invariance analysis, allowing meaningful comparisons. Gender invariance is confirmed at the configural, metric, and scalar levels to guarantee that measurement biases are not the cause of variations in dissociation scores between men and women or between cultural groups. Notably, there were no significant gender differences in dissociation scores (*p* = 0.764, Cohen's *d* = 0.029), suggesting that dissociation affects men and women similarly. This finding is in‐line with Macia et al. [19], who also report strong measurement invariance of the DSS—B across ethnoracial groups in the United States, suggesting that the scale can be used meaningfully across diverse populations. Our study aligns with Macia's research, emphasizing the cultural adaptability of the DSS—B, indicating its ability to provide reliable and valid assessments of dissociation in varied contexts. In our study, variations were seen amongst the nations; nevertheless, the highest dissociation scores were reported by Palestinian participants (*M* = 6.88, SD = 6.81), followed by those from Saudi Arabia, Lebanon, Egypt, and Jordan (*p* < 0.001). Given that the prior research has connected greater dissociation symptoms to trauma type exposure and severity, these discrepancies may also be impacted by sociopolitical and cultural factors. Palestinian participants’ greater dissociation scores are potentially due to the effects of trauma and ongoing stress experienced by the population (see [55]). Lower ratings in Egypt and Jordan, on the other hand, can be the result of less exposure to traumatic events and cultural coping strategies as these two factors are positively correlated with dissociation [56].

The DSS—B demonstrated good concurrent and discriminant validity in addition to its structural validity. The strong correlation between dissociation and psychological distress was confirmed by the significant associations found between dissociation scores and depression‐anxiety (*r* = 0.57, *p* < 0.001), irritability (*r* = 0.51, *p* < 0.001), and loneliness (*r* = 0.45, *p* < 0.001), further supported by research showing a strong link between dissociation and anxiety across adolescents and adults, as well as a significant association with depression in adults with adjustment disorder [57, 58]. Additionally, the bidirectional relationship between dissociation and loneliness suggests that loneliness can trigger dissociation, while dissociation exacerbates loneliness by impairing social connections [59]. Moreover, although the relationship between dissociation and irritability has not been explicitly isolated, findings indicate that PTSD symptom severity—including dissociation—is strongly linked to irritability, suggesting an indirect association between dissociative symptoms and heightened irritability in trauma‐exposed individuals [12]. The AVE value's square root (0.84) exceeded inter‐factor correlations, confirming the scale's capacity to quantify dissociation as a distinct condition. This study adds additional correlations between dissociation and other conditions, similarly to Macia et al. [19], which found that dissociation was significantly associated with PTSD, depression, and alcohol use.

### 4.1 Limitations

Despite this study's advantages, several limitations must be noted. First, the cross‐sectional nature of this study limits the ability to establish causality and prevents us from assessing the test–retest reliability of the scales. Second, data were collected solely through self‐reported measures rather than clinical diagnoses, which may affect the accuracy of responses. Although the measures used have been widely applied in research and shown strong psychometric properties, interpretations should be approached with caution. Further evaluation by mental health professionals is necessary to validate the findings. Third, the study did not include a clinical population, which limits the generalizability of the findings to individuals with diagnosed psychiatric conditions. Future research should replicate this work in clinical settings to assess whether the patterns observed hold in more severe or diagnostically complex samples. Fourth, the use of snowball and respondent‐driven sampling methods may introduce selection biases. Fifth, with 74.5% of participants being female, the sample was primarily young and highly educated, which limits how broadly the results can be applied to the Arabic‐speaking community. Furthermore, even though measurement invariance was verified across nations and genders, further study should be done in Arabic‐speaking areas like the Gulf and North Africa to better understand how dissociative symptoms vary among cultures. To evaluate the stability of DSS—B scores over time, especially in groups exposed to trauma, longitudinal studies are also required. Macia et al. [19] also emphasized the importance of expanding dissociation research across diverse cultural contexts to ensure that assessment tools remain reliable and valid. Like our study, their research calls for continued cross‐cultural validation and further exploration of psychosocial influences on dissociation.

### 4.2 Clinical Implications

Providing a validated Arabic version of the DSS—B equips mental health professionals in Arabic‐speaking countries with a reliable tool for identifying and monitoring dissociative symptoms. This culturally adapted measure enhances diagnostic accuracy and supports early intervention, which is especially critical for individuals with complex trauma histories. Beyond its role in screening, the DSS—B can also be used to evaluate treatment outcomes in dissociation‐focused interventions. Recent studies have highlighted promising therapeutic approaches, such as Dissociation‐Focused Cognitive Behavioral Therapy (DF‐CBT), which demonstrated reductions in dissociative symptoms in patients with the PTSD‐DS [60], and the “Finding Solid Ground” program, which showed effectiveness in stabilizing individuals with complex dissociation through a structured, skills‐based group model [61]. Moreover, emerging evidence from randomized controlled trials suggests that dissociation may influence treatment responsiveness, with higher dissociative symptomatology predicting different outcomes across modalities like DBT‐PTSD and cognitive processing therapy (CPT) [62]. These findings underscore the importance of assessing dissociation when tailoring therapeutic approaches. In‐line with the International Society for the Study of Trauma and Dissociation (ISSTD) guidelines, phase‐oriented models that prioritize stabilization and emotion regulation remain central to the treatment of complex dissociative conditions [63]. The Arabic DSS—B offers a valuable foundation for integrating such interventions into clinical practice, promoting culturally sensitive, data‐informed care across Arabic‐speaking contexts.

## 5 Conclusion

By demonstrating strong psychometric properties, including high reliability, measurement invariance, and validity, this study establishes the DSS—B as an effective tool for assessing dissociation across different cultural contexts. The DSS—B is especially helpful in clinical settings for quick dissociation assessments and in research where dissociation needs to be quantified with other psychiatric conditions because of its concise style and solid scientific basis. These results highlight how crucial cross‐cultural validation is to ensuring that mental health assessment instruments fairly and accurately assess dissociation among various ethnicities. In order to improve the evaluation and comprehension of dissociative symptoms in the Arab world and support more culturally sensitive therapeutic procedures and research, a validated Arabic version of the DSS—B is essential. By aligning with Macia et al. [19], our study further supports the importance of brief, reliable dissociation measures that can be applied across various populations, ensuring wider accessibility and accuracy in assessing dissociative symptoms worldwide.

Future research should focus on further validating the DSS—B across diverse populations, particularly in underrepresented cultural and clinical groups, to enhance its generalizability. Longitudinal studies are needed to assess the stability of dissociation symptoms over time and explore potential causal relationships between dissociation and psychological distress. Additionally, future investigations should integrate clinician‐administered diagnostic tools alongside self‐report measures to strengthen the accuracy of dissociation assessments. Furthermore, expanding research to include neurobiological and physiological markers of dissociation could provide deeper insights into its underlying mechanisms. Lastly, cross‐cultural validation of the DSS—B in non‐Western settings, including other ethnoracial groups, could ensure its applicability in a wider range of clinical and research environments.

## Author Contributions

Feten Fekih‐Romdhane, Souheil Hallit, and Sahar Obeid designed the study. Anthony Rizk and Adella Ibrahim drafted the manuscript. Diana Malaeb, Amira M. Ali, Mirna Fawaz, Nouran Omar El Said, Nisma Merdad, Rizwana Amin, Wizra Saeed, Muna Barakat, and Rami Mosleh collected the data. Souheil Hallit carried out the analysis and interpreted the results. All authors reviewed the article for intellectual content; all authors reviewed the final manuscript and gave their consent.

## Disclosure

The authors have nothing to disclose.

## Ethics Statement

The Institutional Review Board office of Rayak University Hospital granted this study ethical approval (ECO‐R‐410).

## Consent

Written informed consent was obtained from all subjects; the online submission of the soft copy was considered equivalent to receiving a written informed consent. Every step of the process was carried out in compliance with all applicable laws and rules (such as the Declaration of Helsinki).

## Conflicts of Interest

The author declares no conflicts of interest.

## Data Availability

The datasets generated and/or analyzed during the current study are not publicly available but are available from the corresponding author on reasonable request (SH).
